# Fully automated head-twitch detection system for the study of 5-HT_2A_ receptor pharmacology *in vivo*

**DOI:** 10.1038/s41598-019-49913-4

**Published:** 2019-10-03

**Authors:** Mario de la Fuente Revenga, Jong M. Shin, Hiba Z. Vohra, Kelsey S. Hideshima, Matthew Schneck, Justin L. Poklis, Javier González-Maeso

**Affiliations:** 1 Department of Physiology and Biophysics, Virginia Commonwealth University School of Medicine, Richmond, VA 23298 USA; 2 Department of Biomedical Engineering, Virginia Commonwealth University, Richmond, VA 23220 USA; 3 Department of Pharmacology and Toxicology, Virginia Commonwealth University, Richmond, VA 23298 USA

**Keywords:** Biological techniques, Behavioural methods

## Abstract

Head-twitch behavior (HTR) is the behavioral signature of psychedelic drugs upon stimulation of the serotonin 5-HT_2A_ receptor (5-HT_2A_R) in rodents. Following the previous report of a semi-automated detection of HTR based on the dynamics of mouse’s head movement, here we present a system for the identification of individual HTR events in a fully automated fashion. The validity of this fully automated HTR detection system was tested with the psychedelic drug DOI in 5-HT_2A_R-KO mice, and via evaluation of potential sources of false-positive and false-negative HTR events. The increased throughput in data processing achieved via automation afforded the possibility of conducting otherwise time consuming HTR time-course studies. To further assess the versatility of our system, we also explored the pharmacological interactions between 5-HT_2A_R and the metabotropic glutamate receptor 2 (mGluR2). Our data demonstrate the potentiation effect of the mGluR2/3 antagonist LY341495 on DOI-induced HTR, as well as the HTR-blocking effect of the mGluR2/3 agonist and antipsychotic drug in development LY404039. This fully automated system can contribute to speed up our understanding of 5-HT_2A_R’s pharmacology and its characteristic behavioral outputs in rodents.

## Introduction

The study of rodent behavior has experienced in recent years a significant degree of automation, which is consonant with the demand of reproducibility in biomedical research^1^. The outcomes of rodent behavior studies are sensitive to a number of biases emerging from laboratory idiosyncrasies and analyst’s expectations that can affect the output of behavior assessment in traditional scoring by direct observation. As a consequence, elimination of the observer via automation offers both a greater degree of homogeneity in the execution of behavioral techniques and significant increases of data throughput by enabling the processing of larger datasets that would otherwise be unfeasible or extremely time consuming^1,2^.

Classic hallucinogens, also known as psychedelics, such as lysergic acid diethylamide (LSD), psilocybin (and its active dephosphorylated metabolite psilocin), mescaline and (±)-2,5-dimethoxy-4-iodoamphetamine (DOI), induce profound alterations in human perception, cognition, emotion and sense of self^3,4^. In rodents, psychedelics produce a characteristic side-to-side movement of the head commonly known as head-twitch response (HTR). This characteristic behavior is not induced by other psychoactive drugs, such as cocaine, phencyclidine (PCP), scopolamine, or amphetamine, and is thus widely employed to study the behavioral pharmacology of psychedelics in rodent models^5–7^.

Previous findings based on both pharmacological blockade with relatively selective serotonin 5-HT_2A_ receptor (5-HT_2A_R) antagonists, such as volinanserin (M100907) and ketanserin, and genetic deletion of certain G protein-coupled receptors (GPCRs) in knockout (KO) mice have provided convincing evidence that the HTR induced by psychedelics is at least in part mediated via serotonin 5-HT_2A_R-dependent signaling^5,8–13^. Regarding the subjective effects of psychedelics, analogous findings have been observed in healthy volunteers. Thus, administration of the 5-HT_2A_R antagonist ketanserin prevented the psychedelic effects of psilocybin and LSD in humans^14–16^. Our previous work focused on the fundamental paradox that all known hallucinogens, such as LSD, mescaline and psilocybin, are 5-HT_2A_R agonists, but yet closely related 5-HT_2A_R agonists, such as lisuride and ergotamine, do not exhibit hallucinogen activity. We previously reported that HTR is exclusively induced by hallucinogenic 5-HT_2A_R agonists, and not by closely related non-hallucinogenic 5-HT_2A_R agonists, further highlighting the parallelism between the psychedelic drugs’ subjective effect in humans and induction of HTR in rodents^5^. Despite the lack of face validity, the strong correlation between the behavioral characteristics induced by psychedelics among species supports the use of the HTR as a rodent behavioral proxy for human psychedelic action.

A recent report described the use of a magnetometer-based approach for the detection of HTR in mice^17^. This advantageous semi-automated set up shifted the classic and time-consuming scoring of tape-recorded videos to visual analysis of the magnetometer signal for the detection of HTR. Herein, in an attempt to further reduce data processing time of HTR experiments – eliminating also experimenter’s bias, we developed an analogous set up that features a fully automated scoring process by computer-assisted detection of individual HTR events. We compared the automated assessment to other methods of analysis, corroborated our preceding findings in 5-HT_2A_R-KO, and analyzed potential sources of false-positive and false-negative detection. The reduction in processing time increased the throughput of our system, making it suitable for otherwise extremely time-consuming behavioral pharmacology HTR assays, such as time-courses and dose-response curves. Lastly, we further studied the pharmacological crosstalk between the metabotropic glutamate receptor 2 (mGluR2) and 5-HT_2A_R, along with some pharmacokinetic considerations.

## Results

### Automated detection of head-twitch response

**Figure 1:**
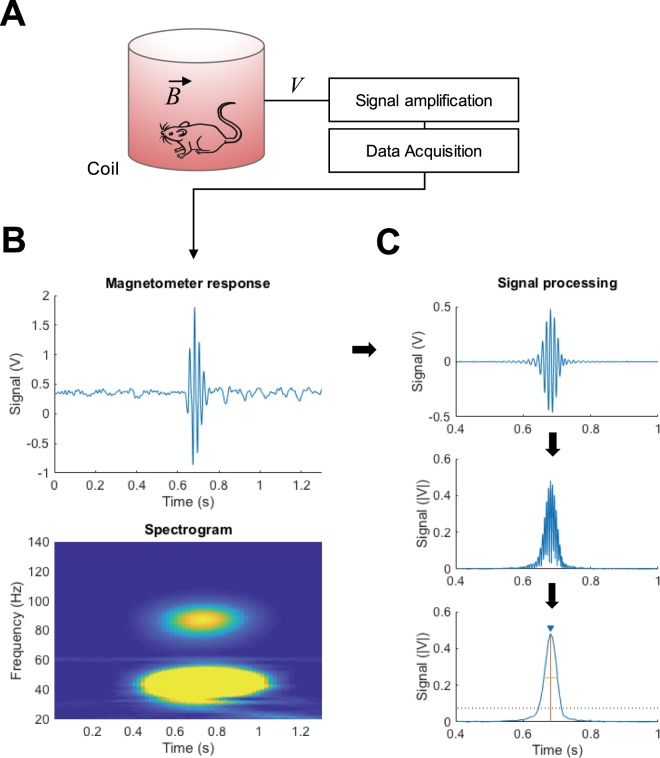
Scheme of the set-up employed (**A**). A small magnet, surgically implanted on the mouse skull surface, produces an electrical signal of greater amplitude than background noise when the mouse displays an HTR. The signal is amplified and transduced by a data acquisition system. Trace of raw data showing an HTR event (**B**, upper panel) and spectrogram corresponding to the HTR event (**B**, lower panel). Note the 80–100 Hz component characteristic of HTR. Scheme of the signal processing and detection system (**C**). Amplified magnetometer signal band-pass filtered between 70–110 Hz (**C**, upper panel), transformation to absolute values (**C**, mid panel), and double local maxima processing for detection of individual HTR events (**C**, lower panel). An individual HTR event is registered (inverted closed blue triangle) when the peak prominence (vertical orange line) exceeds the amplitude threshold (dotted black line) and meets the requirement of width (horizontal yellow line) and separation from adjacent peaks (not shown). See also Fig. S1.

Following a previous report by Halberstadt and Geyer^17^, we assembled a set-up for the magnetometer-based detection of HTR (Fig. 1). Mice with a small neodymium magnet surgically implanted on the top of the cranium were placed in a coiled chamber where the movement of the head was converted to voltage signals by electromagnetic induction. The raw signal was passed through a preamplifier to be then transduced and recorded by a data acquisition system (Fig. 1A). Consistent with the previously described semi-automated magnetometer detection system of HTR^17^, visually identified HTRs on video tape were registered by the magnetometer as sinusoidal waves of significant amplitude over background noise with two frequency components in the 40–50 Hz and 80–100 Hz range (Figs 1B and S1). No other behavioral events commonly observed in mice, such as grooming, rearing, sniffing or ambulation, overlapped with strong maxima in the higher frequency band (80–100 Hz) of the spectrogram. In light of this, we developed a signal processing script on MATLAB for the detection of individual events in the high frequency range (80–100 Hz, Fig. 1C).

Briefly, data was band-passed between 70–110 Hz, and, after transformation to absolute values, local maxima were extracted. This transformation, equivalent to a wave envelope, resulted in a smoothening of the signal such that wavelets in the raw data became individual unipolar peaks (Fig. 1C, lower panel trace). Conditional detection of new maxima after local maxima extraction was employed for the identification of individual HTR events (Fig. 1C, lower panel annotations).

The amplifier delivered a constant gain value throughout the 10–200 Hz range for input signal amplitudes below 15 mV (Fig. S2). We found that the raw coil signal amplitude in response to animal exhibiting HTR was <15 mV (data not shown). Exceeding the 15 mV threshold could result in a distorted amplified signal and impact the performance of the detector. Therefore, should the system undergo substantial modifications (*i.e*. magnet strength, coil turns) monitoring the coil output is advised to avoid these potential issues.

### Automated system validation

The conditional detection of individual HTR was based on peak maximum width (*i.e*., duration of the event), minimum separation from other events, and peak prominence (see a graphic representation on Figs 1C and S1). Preliminary studies demonstrated that automated HTR detection was the most sensitive to peak prominence (data not shown), as the other two filters (width and separation) were by large more permissive. The prominence threshold was set to 15 standard deviations of the 70–110 Hz band-passed signal with a top limit of 0.075 V (Fig. 1C, dotted line). The threshold and the limit were chosen based on optimal linear regression of fully automated detection of HTR vs. visual count assisted with the corresponding spectrogram on 100 datasets (Fig. S3, F[1,98] = 31,599).

**Figure 2:**
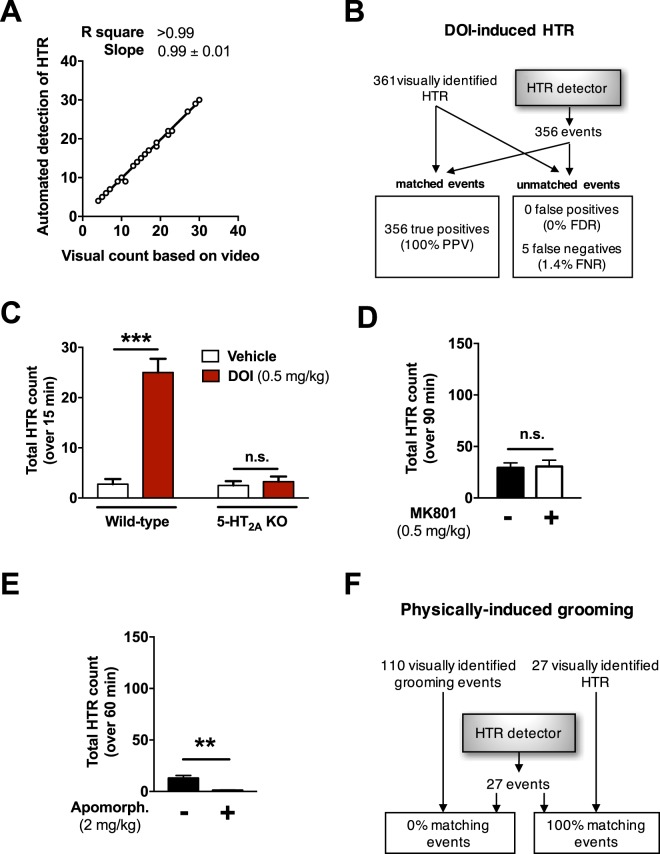
Validation of the automated detection set-up. (**A**) Correlation between automated detection and visual count of HTR on 10-week-old mice treated with different doses of DOI. (**B**) Graphical scheme of the experiment and evaluation of the detector performance relative to visually-detected HTR. (**C**) The magnetometer system is able to detect robust HTR after administration of DOI (0.5 mg/kg, i.p.) in wild-type, but not in 5-HT_2A_ KO mice (n = 4 per group). (**D**) HTR count over a period of 90 min in control mice treated with MK801 (0.5 mg/kg, i.p.) or vehicle (n = 6 per group). (**E**) HTR count over a 60 min period in control mice administered apomorphine (2 mg/kg, i.p.) or vehicle (n = 4 per group). (**F**) Schematic representation of the discriminative power of the HTR detector in mice (n = 3) showing HTR and grooming behavior induced physically by sprinkling fine wood chips on the head of the animal. Linear regression (**A**,**B**, slope value ± S.E.M.). Two-way ANOVA with Bonferroni’s *post hoc* test (**C**). Two-tailed Student’s *t*-test (**D**,**E**). Values represent mean ± S.E.M. (n.s., not significant, **P < 0.01, ***P < 0.001). PPV: positive predictive value, FDR: false discovery rate, FNR: false negative rate.

To further test the performance of the system, we tested the automated detection of HTR vs. visually detected videotaped HTR on 24 datasets from young adult mice (∼10 week-old-mice) that received different doses of DOI (Fig. 2A, F[1,22] = 5,143). We found a high correlation between both scoring techniques and almost identical absolute values per analyzed dataset (see R square and slope values in Fig. 2A). Further analysis of individual event timestamps revealed that 1.39% of the visually-identified HTR were not detected (Fig. 2B). Importantly, no false positives were identified in these datasets (Fig. 2B).

Based on previous findings suggesting behavioral dysfunctions related to aging in mice^18,19^, we performed another visual vs. automated correlation of HTR detection employing 23 datasets in aged animals (∼12 months of age) treated with different doses of DOI (Fig. S4, F[1,24] = 1,649). The slope value, inferior to 1, suggests that in this cohort of older mice the automated detection is slightly skewed toward false negative errors. Accordingly, the analysis of the individual events detected visually and automatically revealed a false discovery rate of 1.2% and a false negative rate of 9.4%. In spite of the false negative skewness in this cohort of aged mice, the results obtained using both methods (*i.e*., visual vs. fully automated) were highly correlated (R square >0.99), which supports the representativeness of automated detection. Further visual analysis showed that HTR in aged mice results in a signal of lower amplitude when compared to younger mice, suggesting an overall “weaker” HTR in the older cohort.

In order to further validate our system and the automated detection script, we aimed to test the 5-HT_2A_R-dependance of HTR in 5-HT_2A_-KO mice and controls (Fig. 2C). Following our previously described protocol^5^, mice were injected (i.p.) with saline or DOI (0.5 mg/kg). Right after injection, mice were placed in the magnetometer for 30 min, and recorded for the last 15 min. As expected, DOI induced a robust HTR effect in wild-type mice that was not paralleled by 5-HT_2A_-KO littermates (Two-way ANOVA: Genotype effect F[1,12] = 31.15, *P* < 0.001; Treatment effect F[1,12] = 38.21, *P* < 0.001; *post hoc*: Wild-type (Saline vs. DOI), *P* < 0.001; *5-HT*_*2A*_*-KO* (Saline vs. DOI), *P* > 0.05).

Sub-anesthetic doses of non-competitive NMDA receptor antagonists, such as phencyclidine (PCP) and MK801, induce a pronounced and sustained hyperlocomotor activity in mice and rats^20,21^. In order to assess whether a significant increase of motor activity could affect the HTR count of the detector, we aimed to test the effect of a low dose of MK801 alone on HTR count. Mice were habituated to the coil for 30 min and then administered (i.p.) with MK801 (0.5 mg/kg), or vehicle. Immediately after drug administration, mice were recorded in the magnetometer for 90 min. HTR were quantified for each treatment condition for the total duration of the experiment (Fig. 2D). The summation of all HTR events for the 90 min duration test revealed no differences between MK801 or saline (*P* > 0.05). Despite the lack of differences in HTR events between both treatment groups, we observed in the periodogram of the raw data an increase in the frequency range 25–30 Hz in the MK801-treated group of mice suggestive of low frequency motor events in the drug-treated animals that could result as a consequence of the effect of MK801 on hyperlocomotor activity (Fig. S5).

Apomorphine is a dopaminergic agonist that also behaves as a weak 5-HT_2A_R antagonist^22^, known to induce in rodents stereotyped behavior such as sniffing, rearing, gnawing and climbing^23^. In order to assess the performance of the HTR detector on mice exhibiting stereotyped behaviors, we tested our HTR automated detection system against mice treated with apomorphine (Fig. 2E). After a 30 min habituation period, mice were administered apomorphine (2 mg/kg, i.p.) or vehicle, and recorded for additional 60 min. Relative to vehicle, HTR in the apomorphine-treated group was slightly but significantly reduced (Fig. 2E, t6 = 4.182, *P* = 0.0058). The 5-HT_2A_R antagonist nature of apomorphine is likely responsible for the suppression of spontaneously occurring HTR. Still, but more importantly, this finding also demonstrates that the dopamine-mediated stereotypies induced by this apomorphine are not erroneously triggering HTR detection.

To further test for an independent source of false positive detection, we assessed the performance of the detector against grooming, a typical self-care behavior in rodents. As expected based on previous findings employing a water spray^24^, grooming behavior was easily induced by gently sprinkling fine size wood chips on animal’s head. Along with 27 HTR, 110 grooming events were visually detected during a period of 2–5 min per mouse (n = 3). The matching of timestamps showed that all 27 visually-identified HTR corresponded to the 27 HTR events annotated by the HTR detector. More importantly, none of the visually identified grooming events were falsely identified as HTR (Fig. 2F).

Certain dopamine D_1_ receptor agonists, such as SKF38393, are known to induce both strong and sustained grooming bouts as well as a mild increase in HTR events^17,25^. To further assess the performance of the detector in drug-induced aggressive grooming, mice (n = 3) were administered with SKF38393 (10 mg/kg, i.p.), and the signal was recorded for 10 min at the 20 min time point after administration of the drug. Importantly, the timestamps corresponding to the 22 HTR identified by the detector matched with the 22 events that were visually-identified, and not with grooming bouts. Additionally, the number of HTRs identified in the SKF38393 treatment group was numerically superior but statistically comparable to the vehicle-treated group of mice (5.0 ± 0.5 (vehicle) vs. 7.3 ± 2.4 (SKF38393), t_4_ = 0.944, P = 0.39).

Jumping is observed in escaping behavior from a number of mouse strains commonly used in research. Although jumping behavior does not seem to be induced by classic psychedelic 5-HT_2A_R agonists in mice^5^, the fast acceleration during this event would result in an abrupt change in the coil signal that could falsely trigger HTR detection. To test this possibility, we first studied the performance of the HTR detector in mice exhibiting jumping behavior as a consequence of naloxone-precipitated withdrawal. We adapted a previously described protocol^26^ that consists of a single administration of the μ-opioid receptor agonist morphine (50 mg/kg, i.p.) to control mice (n = 3) 4 h prior to the administration of the μ-opioid receptor antagonist naloxone (10 mg/kg, i.p.). Jumping events were recorded during the 15 min following naloxone administration. Importantly, out of 66 visually identified jumping events, 38 were identified as false positive by the HTR detector, yielding a false positive detection rate of 57.6% (42.4% true negative discrimination rate).

**Figure 3:**
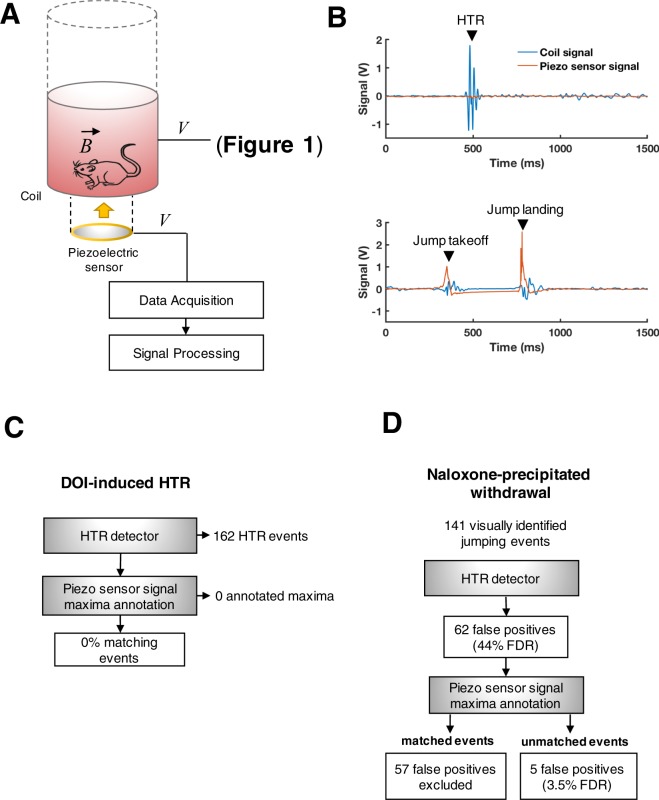
Scheme of the set-up and evaluation of automated HTR detection combining magnetometer and piezo sensor signal analysis. (**A**) Depiction of the set-up employed for the simultaneous recording of the magnetometer and piezo sensor signals. (**B**) Upper panel, traces corresponding to an HTR event; lower panel, traces corresponding to a jumping event recorded by both systems simultaneously. (**C**) Matching analysis scheme for events detected by the automated HTR detection system and the piezo sensor in control mice (n = 3) exhibiting HTR induced by DOI (1 mg/kg, i.p.). (**D**) Graphical depiction of the HTR detector false positive detection rate (FDR) when used alone, or in combination with piezo sensor signal maxima matching, in mice (n = 3) exhibiting jumping behavior during naloxone (10 mg/kg, i.p.)-precipitated withdrawal (morphine 50 mg/kg, i.p., 4 h prior).

Based on these pilot data, we next attempted to develop a secondary system based on a piezoelectric sensor to improve HTR detection by filtering out potential jumping events – more specifically by evaluating the changes in pressure at the bottom of the coil container as a consequence of mouse jumping behavior (Fig. 3A). We first compared the coil and piezo sensor signal in response to HTR and jumping events (Fig. 3B). DOI-induced HTR produced the characteristic wavelet described above in the coil signal, along with very low amplitude disturbances – almost undistinguishable from the baseline piezo sensor signal (Fig. 3B, upper panel). Jumping observed during naloxone-induced withdrawal, however, produced irregular wavelets in the coil signal that coincided with spikes in the voltage output of the piezo sensor (Fig. 3B, lower panel). The signals on either sensor were found to correspond to the takeoff and landing of jumping events. Consequently, we designed a simple algorithm to identify maxima in the piezo sensor signal exceeding an absolute value of 0.3 V for annotating potential jumping events. The threshold was determined experimentally based on the maxima values observed during jumping events relative to the baseline signal.

To assess whether this algorithm interferes with HTR detection and to further corroborate the abovementioned observation that HTR does not produce substantial changes in the piezo sensor signal, we tested the piezo sensor signal annotated maxima against automated HTR detection in mice exhibiting DOI-induced HTR. For this purpose, mice (n = 3) were administered DOI (1 mg/kg) and the outputs of the coil and piezo sensor were simultaneously recorded for the following 15 min. The coil signal was analyzed by the HTR detector, and the timestamps of the corresponding HTR events matched with the annotations of maxima in the piezo signal (Fig. 3C). As expected, none of the 162 HTR events identified by the HTR detector had a match in the piezo sensor annotated maxima, supporting the notion that the piezo sensor signal analysis did not interfere with the automated detection of true HTR events.

Considering the lack of interference of the piezo sensor signal analysis on HTR detection, we next aimed to evaluate the discrimination power resulting from combining the automated HTR detector and piezo sensor annotated maxima for the exclusion of potential jumping events during naloxone-precipitated withdrawal. The coil and piezo sensor signal were recorded in control mice (n = 3) that were also videotaped for 15 min following the administration of naloxone. Similar to the prior pilot experiment described above, out of 141 visually identified jumping events, the HTR detector falsely identified 62 as HTRs (Fig. 3D). Then, the annotated piezo sensor signal maxima were matched to the timestamps of the HTR detector. Out of the 62 jumping events that triggered false positive detection in the HTR detector, 57 were found to match maxima on the piezo sensor signal and were hence excluded. Consequently, and importantly, the combination of the HTR detection and piezo sensor maxima matching analysis resulted in a reduction of the false discovery rate to 3.5% (Fig. 3D).

### Relationship between DOI pharmacokinetics and HTR

**Figure 4:**
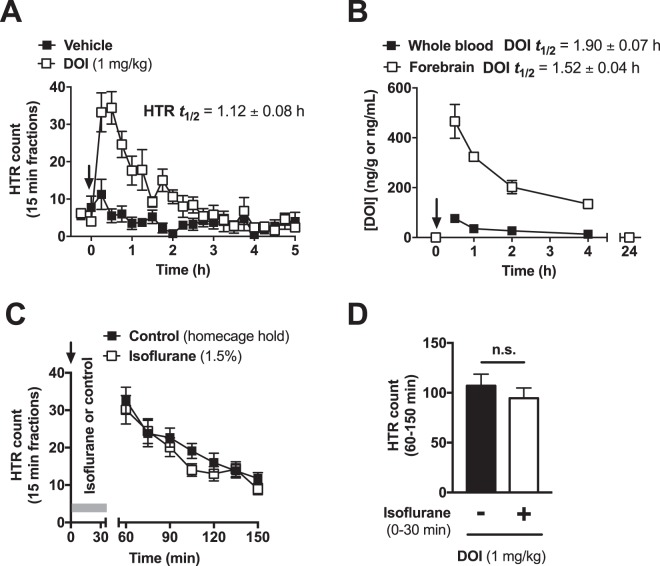
Time-dependent effect on HTR and disposition profile of DOI. (**A**) HTR induced by DOI (1 mg/kg, i.p.) along with time after the administration of the drug (n = 5) or vehicle (n = 4). (**B**) Temporal distribution of DOI (1 mg/kg, i.p.) in mouse whole blood and forebrain (n = 4 per time point and tissue). (**C**) HTR time-course on mice treated with DOI (1 mg/kg, i.p.) put under isoflurane anesthesia (1.5%, n = 10) or held in the home cage (n = 9) for the first 30 min following administration of the drug. (**D**) Quantification of total HTR count between 60–150 min. Two-tailed Student’s *t*-test (**D**). Values represent mean ± S.E.M. (n.s., not significant). Arrow indicates time of administration of DOI or vehicle.

Halogenated phenethylamines, such as DOI or 2,5-dimethoxy-4-bromophenethylamine (2-CB), are known to have long lasting psychedelic effects in humans and low clearance from rat brain^27–29^. To further explore the convenience of our system for studying early and delayed behavioral effects of 5-HT_2A_R stimulation, we performed a DOI-induced HTR time-course study (Fig. 4A). Mice were habituated to the coil for 30 min, after which they were administered (i.p.) with DOI (1 mg/kg) or vehicle and recorded for 5 h. DOI induced a rapid and pronounced increase in HTR count (Two-way ANOVA: Time effect, F[19,33] = 10.77, *P* < 0.001; Treatment effect, F[1,7] = 28.61, *P* = 0.0011).

HTR induced by DOI peaked during the first 15 min fractions, after which the HTR count progressively returned to basal levels. In light of this, we aimed to fit HTR as a function of time to an exponential decay equation: $HTR(t)=HT{R}_{15min}\cdot {e}^{-\lambda t}$, where *HTR*(*t*) is the HTR as function of time in 15 min bins, *HTR*_15*min*_ is the HTR count in the first 15 min bin and *λ* is the rate of effect decay. The parameter *λ* was extracted as slope from linear regression applied to the natural logarithmic values of *HTR*(*t*) (Fig. S6, $ln[HTR(t)]=ln[HT{R}_{0}]-\lambda t$). HTR effect half-life was calculated as $HT{R}_{{t}_{1/2}}\,=\,ln2/\lambda$ (Fig. 4A)^30^.

We next aimed to study the pharmacokinetics of DOI to assess whether the decay of HTR after intraperitoneal administration of DOI was correlative to the disposition of the drug. The concentration of DOI was determined in systemic blood and forebrain harvested from a separate group of mice sacrificed at different time-points after administration of DOI (1 mg/kg, i.p.). As anticipated from the HTR time-course, the absorption of the drug was rapid, and reached its maximal concentration during the first 30 min in both tissues. Interestingly, the maximal concentration of DOI was several-fold greater in forebrain suggesting accumulation of DOI could be occurring in this tissue. The half-life of the drug was calculated as $DO{I}_{{t}_{1/2}}=ln2/\lambda$ (Fig. 4B), after fitting the concentration of DOI in whole blood and forebrain to the exponential decay equation $ln[DOI](t)=ln{[DOI]}_{30min}-\lambda t$ (Fig. S7) from which *λ* was extracted. The time-course of DOI disposition in mice whole blood and forebrain (Fig. 4B) is qualitatively similar to the rate of decay of HTR (Fig. 4A). The Pearson´s correlation coefficient between HTR counts and the concentration of DOI in different tissues at the matched time-points further corroborated this notion (HTR vs. whole blood [DOI], Pearson’s *r* = 0.990, *P* = 0.010; HTR vs. forebrain [DOI], Pearson’s *r* = 0.990, *P* = 0.010).

In spite of this positive correlation between drug disposition and observed effects, the half-life of DOI in forebrain is 36% longer (∼24 min) than that of HTR decay (Fig. 4A,B, above). In light of this, we next aimed to determine whether the shorter half-life of DOI-induced HTR could be related to muscular fatigue resulting from the high frequency of HTR events that follow shortly after administration of the drug. To assess this, we used isoflurane anesthesia (1.5%) to block all motor activity for the first 30 min following administration of DOI (1 mg/kg, i.p.). The control group was held in the home cage for the 30 min right after the administration of DOI. Previous findings by other groups have reported that jerks and twitches may be still observable in DOI (∼4 mg/kg)-treated mice under isoflurane anesthesia^31^. In our case, no motor activity was evident, other than respiratory function, in DOI (1 mg/kg)-treated isoflurane-anesthetized mice. After the initial 30 min anesthesia or home-cage hold phase, the animals were placed in the magnetometer where the anesthetized animals were allowed to recover during the 30–45 min epoch. Full recovery of gait and motor function was apparent after about 5 min. Interestingly, the previously anesthetized animals displayed a HTR time-course comparable to that of control mice (Fig. 4C,D, t_7_ = 0.289, *P* > 0.05), suggesting that fatigue is probably not responsible for the drop in HTR counts that follows the intense HTR observed during the first 30 min after administration of DOI (see Fig. 4A).

### Modulatory action of mGluR2/3 on HTR

**Figure 5:**
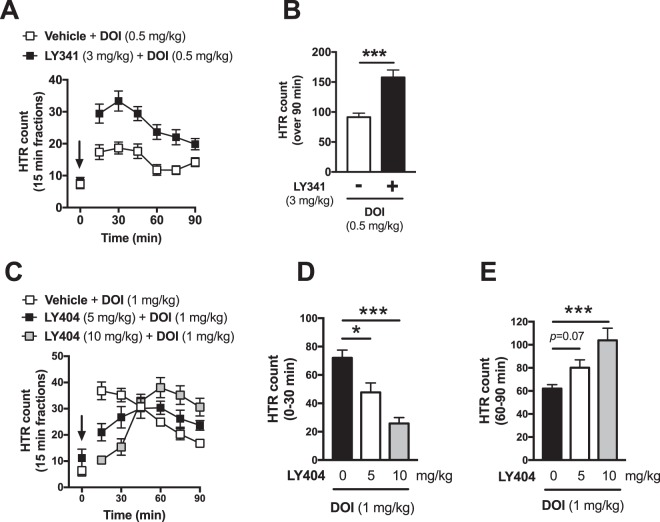
Modulation of DOI-induced HTR by mGluR2/3. (**A**) Effect of the mGluR2/3 antagonist LY341495 (3 mg/kg, i.p.) on DOI (1 mg/kg, i.p.)-induced HTR (n = 14); (**B**) quantification of total HTR count for the 90 min following administration of the DOI. (**C**) Effect of vehicle (n = 11) and different doses of mGluR2/3 agonist LY404039 (5 mg/kg (n = 11) and 10 mg/kg (n = 5) on DOI (1 mg/kg, i.p.); quantification of total HTR count between 0–30 min (**D**) and 60–90 min (**E**) after the administration of DOI. Two-tailed Student’s *t*-test (**B**), one-way ANOVA with Bonferroni’s *post hoc* test (**D**,**E**). Values represent mean ± S.E.M. (*P < 0.05, ***P < 0.001). Arrow indicates time of administration of DOI.

Based on previous reports^32,33^, including ours^8,20,34,35^, showing the modulatory action of mGluR2 on 5-HT_2A_R-mediated responses, we next aimed to study the effect of drugs targeting the mGluR2 on DOI-induced HTR. Mice were allowed to habituate to the coil environment for 15 min, after which they were administered the mGluR2/3 antagonist LY341495 (3 mg/kg) or vehicle (i.p.). After a 30 min recorded lag, mice were administered DOI (0.5 mg/kg) and recorded for additional 90 min (Fig. 5A). Compared to vehicle-treated mice, LY341495 alone failed to induce any significant changes in the HTR count over the 30 min lag period (Fig. S8, t_26_ = 0.913, *P* > 0.05). However, after administration of DOI, LY341495 significantly increased the DOI-induced HTR time-course (Fig. 5A), and total count in the 90 min phase following DOI administration (Fig. 5B, t_26_ = 4.293, *P* < 0.001).

We next aimed to study the effect of the orthosteric mGluR2/3 agonist LY404039 (LY404) on DOI-mediated HTR in our automated system. Mice were allowed to habituate to the coil for 15 min, after which they were administered the mGluR2/3 agonist LY404039 (5 mg/kg, 10 mg/kg) or vehicle (i.p.), followed by the administration of DOI (1 mg/kg) 5 min after. Mice were recorded for additional 90 min (Fig. 5C). At early time points (<45 min after administration of DOI), LY404039 induced a dose-dependent attenuation of DOI-induced HTR response (Fig. 5D, one-way ANOVA: F[2,24] = 11.14, *P* < 0.001; *post hoc*: DOI(veh. vs. LY404 5 mg/kg), *P* = 0.012, DOI(veh. vs. LY404 10 mg/kg), *P* < 0.001). Unexpectedly, after the initial attenuation effect, at later time points (>45 min), a LY404039 dose-dependent potentiation of HTR occurred (Fig. 5C,E, one-way ANOVA: F[2,24] = 8.56, *P* = 0.002; *post hoc*: DOI(veh. vs. LY404 5 mg/kg), *P* = 0.068, DOI(veh. vs. LY404 10 mg/kg), *P* < 0.001).

## Discussion

Unlike other paradigms employed in the study of psychoactive drugs, such as pre-pulse inhibition (PPI) of the startle or changes in locomotor activity, HTR bears a great degree of receptor specificity that is uncommon in the study of GPCR-mediated behavioral pharmacology^5^. Not only HTR is a consequence of 5-HT_2A_R activation in rodents, but it is also predictive of psychedelic effects in humans and can be employed to discriminate psychedelic from known non-psychedelic 5-HT_2A_R agonists^5,7^. Herein we presented and validated a fully automated HTR detection system based on a previously reported semi-automated magnetometer set up for the study of the 5-HT_2A_R behavioral pharmacology^17^.

To our knowledge, the first study reporting mouse HTR behavior induced by the psychedelic drug LSD was published by Keller and Umbreit in 1956^36^. However, it was not until mid-2000s with our previous observations suggesting the potential use of HTR as a mouse behavioral proxy of human psychedelic potential^5^ that this particular behavior became routinely used in molecular and pharmacological studies related to 5-HT_2A_R-dependent behavioral events. Nevertheless, this method typically requires the visual scoring of videotapes for head-twitches by experienced observers. This protocol is tedious, and in practice it is often variable from one practitioner to another. Most recently, a semi-automated HTR system was developed for detecting HTR based on visual analysis of the magnetometer signal. Although advantageous, this previously developed semi-automated HTR detection system still required manual assessment procedures and hence it may not be suitable for high-throughput data production. The new automated data processing script demonstrated comparable performance to visual signal analysis and video-based visual assessment. Thus, the two main advantages of our fully automated HTR system presented here are its remarkable reduction of data processing time, and the elimination of visual quantification.

We also show that common mouse behaviors such as motor activity and grooming do not affect the number of HTR events recorded by the fully automated HTR system. Nevertheless, it is worth mentioning that, although the detector showed remarkable equivalence to visual assessment of HTR under the conditions and drugs tested here, the use of our current fully automated system does not preclude the necessity of performing additional observations or behavioral tests to control for false positive-triggering events; especially in the case of new drugs and mouse lines prone to motor abnormalities such as jumping behavior and seizures. In the particular case of jumping behavior, we showed here that the performance of the HTR detector alone is greatly limited by its high degree of false positive detection. With this in mind, we also showed that the addition of a secondary filter for false positive exclusion based on the signal of a piezoelectric sensor greatly reduced the false discovery rate to a statistically acceptable level (*i.e*., less than 5% of false positives). In our previous experience on DOI-induced HTR behavior in mouse strains such as 129S6/SvEv^5^, CD-1^37^ and C57BL/6^38^, the occurrence of jumping is extremely rare, particularly when the testing environment deters escaping behavior. Nonetheless, the combination of HTR detection with correction by piezo sensor maxima matching analysis may be necessary when testing known classic psychedelics or new potential 5-HT_2A/2C_R ligands so long as the occurrence of escaping behavior cannot be ruled out.

This fully automated HTR detection system also allowed us to perform HTR time-course studies with DOI, a drug known to exert long-lasting psychedelic effects in humans^27^. Relative to other psychedelics characterized in mice with much shorter effects on HTR, such as LSD^17^ or 5-methoxy-*N*,*N*-dimethyltryptamine^39^, our study shows that DOI also displays a rather sustained effect on HTR-induction. Encouraged by this finding, we characterized the pharmacokinetic profile of DOI in whole blood and forebrain. By comparison of the half-lives, we observed a faster extinction of HTR relative to DOI disposition in the brain. The comparable HTR counts observed in mice anesthetized during the first 30 min and mice allowed to freely move in their home cage suggests that the potential fatigue subsequent to persistent head-twitching during the first 30 min is not the mechanism underlying the difference in the decay rates. The faster return of HTR to baseline could be explained by levels of DOI in the brain falling below HTR-inducing threshold – even if they are still above the LOQ. Additionally, psychedelic drugs in general, and DOI in particular, are known to induce down-regulation of 5-HT_2A_R and rapid behavioral tolerance^40,41^, therefore phenomena associated with receptor desensitization cannot be excluded. Notwithstanding the subtle difference in the half-lives of the drug and its effects on HTR, a tight correlation exists between the decay rate of HTR and the concentration of the DOI in blood and brain. We are confident that the evolution of HTR along time is a good approximation to the magnitude of DOI disposition. Interestingly, it was recently reported that the subjective effects of psilocybin in healthy volunteers were in close correlation with both the concentration of psilocin in plasma and occupancy of 5-HT_2A_R in the brain^42^. Although to the best of our knowledge direct comparisons of drug levels and HTR in mice are not available, previous literature supports the predictive value of HTR time-course on the underlying pharmacokinetics of LSD in mice. A great share of [^14^C]LSD disposition occurs within the first 30 min following administration of the drug^43^, similarly HTR greatly decreases during the same time period^17^. The case of 5-methoxy-*N,N*-dimethyltryptamine would be dissonant: at comparable doses (10 mg/kg) and administration route (i.p.) complete extinction of HTR occurs within the first 30 min^39^, while the drug in serum return to baseline levels at times superior to 3 h^44^.

The 5-HT_2A_R is fundamental in the mechanism of action of psychedelic drugs, however other GPCR systems can modulate its behavioral output. We employed our system to study pharmacological interactions between mGluR2 and 5-HT_2A_R *in vivo*. It was previously shown that the mGluR2/3 antagonist/inverse agonist LY341495 positively modulated HTR in rats^33^, but only showed a trend in CD-1 mice^32^. In the present report, we demonstrate that the ability of LY341495 to induced a robust potentiation of DOI-induced HTR is also extensive to C57BL/6 mice. We and others previously demonstrated the ability of orthosteric mGlu2/3 agonists LY354740 and LY379268 to reduce DOI-induced HTR in mice^8,45^, an antipsychotic-like effect likely mediated via mGluR2^20,34^. Accordingly, the mGluR2/3 agonist LY404039 (pomaglumetad, or DB103 under Denovo Biopharma license), and potential antipsychotic in developmental stages, blocked the effect of the psychedelic drug DOI on HTR. LY404039 adds up to the list of mGluR2/3 agonists that block HTR induction suggesting that ability to ablate the HTR-induced by psychedelics is an intrinsic property of mGluR2/3 agonists. The effect of LY404039 on HTR-blockade dissipated quickly, possibly due to differences in the pharmacokinetics of DOI and LY404039. In humans, LY404039 is almost completely eliminated 8–12 h after oral administration, while the psychedelic effects of DOI seem to be persistent after 16 h and beyond^27,46^. These values cannot be extrapolated to mice, but do support the notion that disposition of LY404039 is likely more rapid than that of DOI. Atypical antipsychotics commonly used for the treatment of schizophrenia as well as pimavanserin, recently approved for the treatment of psychosis secondary to Parkinson’s disease, are potent 5-HT_2A_R antagonists/inverse agonists able to block HTR^47,48^. As demonstrated here and on our previous reports, novel therapeutic strategies based on glutamatergic modulation also display HTR-blocking effects that could make our system suitable for phenotypical screening of antipsychotic activity in drug candidates, potentially with the added advantage of offering also preliminary insights on the drug pharmacokinetics.

The G_i/o_-coupled mGluR2 and G_q_-coupled 5-HT_2A_R have been found forming multimeric complexes that affect signaling of each receptor^8,20,35,49^. More precisely, it was previously demonstrated *in vitro* that mGluR2/5-HT_2A_R heterocomplex-mediated changes in G_i/o_ and G_q_ activity could predict the psychoactive effects of compounds that interact with either receptor^20^. Our results validate the behavioral output of mGluR2/5-HT_2A_R interaction predicted *in vitro*, and support the notion that 5-HT_2A_R and mGluR2 engage in an antagonistic cross-talk whereby activation or blockade of mGluR2 affects the behavioral output of 5-HT_2A_R activation in an oppositional manner. LY341495 augmentation of DOI-induced HTR and LY404039 suppression of DOI-induced HTR is easily interpreted under this theoretical framework. The brief effect of the latter is consonant with the pharmacokinetic considerations abovementioned. However, explaining the increase in HTR counts in LY404039-treated mice at longer time-points poses a greater challenge. As discussed above, down-regulation of 5-HT_2A_R follows the activation of this receptor by psychedelics^40^. Additionally, the signaling and behavioral output of 5-HT_2A_ appears to be governed by balance between G_i/o_ and G_q_ when co-expressed with mGluR2^20^. LY404039-mediated activation of mGluR2 could induce changes in the trafficking of either receptor, and/or an imbalance in the population of receptors coupled to G_i/o_ and G_q_^5,20,35^. Upon clearance of the mGluR2 agonist, and in the presence of remaining levels of DOI, such compensatory mechanism could become apparent in the form of increased 5-HT_2A_R signaling and ultimately HTR.

Preliminary clinical studies describing the ability of psychedelics to provide long-lasting therapeutic effects on depression and anxiety^50^, in addition to recent reports on their ability to modulate synaptic plasticity^51^, have renewed the interest of the scientific community in these traditionally neglected and heavily controlled drugs^52^. Despite the inherent limitations of using a paroxysmal head movement in rodents as a surrogate of psychedelic effect in humans, the HTR paradigm affords a complete neuropsychopharmacological framework that integrates molecular drug-receptor interactions and its behavioral manifestation in mice^7^. The ease of use and robustness of the detector-coupled system that we present here could aid in better understanding the pharmacology of 5-HT_2A_R, assess the effects of proximal and distal modulation of 5-HT_2A_R behavioral output by other receptor systems, and the neurocircuitry upstream and downstream of 5-HT_2A_R. Unraveling the complex pharmacology of 5-HT_2A_R behind the effect of psychedelic drugs in rodents could afford valuable information under the prospective possibility of psychedelics being employed in human subject for therapeutic purposes. As discussed above, some of the limitations of the detector relative to false positive detection can be addressed by using complementary behavioral paradigms and/or customarily adapting the detection parameters depending on how potential confounding factors affect the discrimination rate.

## Conclusions

The fully automated HTR detection system presented here provides a robust and powerful tool for studying both 5-HT_2A_R behavioral pharmacology as well as time-dependent events that would otherwise be impractical or extremely time consuming. We have also demonstrated the versatility of our set up for the study of direct ligand-5-HT_2A_R interactions as well as modulatory events via mGluR2 cross-talk. Considering the therapeutic interest of both receptors, we believe that our set-up could be useful in the phenotypical profiling of antipsychotic drug candidates and for better understanding the pharmacology of the 5-HT_2A_R and related GPCR systems.

## Methods

### Animals

Experiments were performed on adult (10–20 weeks old) C57BL/6 male mice (Taconic), unless otherwise stated. Animals were housed in cages with up to 5 littermates at 12 h light/dark cycle at 23 °C with food and water *ad libitum*, except during behavioral testing. Experiments were conducted in accordance with NIH guidelines, and were approved by the Virginia Commonwealth University Animal Care and Use Committee. All efforts were made to minimize animal suffering and the number of animals used.

Experiments involving the use of 5-HT_2A_R knockout (5-HT_2A_-KO) mice were performed on adult (10–20 weeks old) 129S6/SvEv male mice. 5-HT_2A_-KO and controls in these experiments denote *Htr2a*^−/−^ and *Htr2a*^*+/+*^ individuals, respectively, born to heterozygote (*Htr2a*^+/−^) breeders^5^.

Surgical implantation of the magnet was adapted from a previously reported protocol^17^. Mice were anesthetized using a ketamine and xylazine (120 mg/kg and 12 mg/kg, respectively). A circular incision was performed to remove part of the scalp. Membranous tissue in and around the area was repeatedly cleaned with H_2_O_2_ and dried until the bone surface was exposed. Dental cement was applied in the exposed area to attach a small neodymium magnet (6.0, 6.0, 1.5 mm, 490 mg) with the south pole facing the bone surface. The implant remained exposed with the dental cement bordering the skin. The mice were allowed to recover for 1 or 2 weeks. Animals were used repeatedly with a washout period of at least 1 week between tests.

Isoflurane anesthesia was rapidly induced at an initial dose of 2% (vol/vol). When the effects of the anesthesia were apparent the animals were quickly administered DOI (1 mg/kg, i.p.) and placed on a heating pad on the isoflurane chamber. The dose of isoflurane was maintained at 1.5% for the following 30 min. Animals were regularly monitored.

### Drugs

(±)-2,5-Dimethoxy-4-iodoamphetamine hydrochloride (DOI), (+)-MK801 hydrogen maleate (dizocilpine), and *R*-(−)-apomorphine hydrochloride hemihydrate, morphine sulphate pentahydrate and naloxone hydrochloride dihydrate were purchased from Sigma Aldrich; LY341495 disodium salt and (±)-SKF38393 hydrobromide were purchased from Tocris. LY404039 (pomaglumetad) was purchased from Selleckchem. All drugs were dissolved in saline (0.9% NaCl) to the appropriate volume (0.005–0.01 ml/g body weight) and concentration for administration (i.p.). Vehicle-treated condition denotes injection of saline (i.p.) to the equivalent volume of the drug administered.

### Materials

Data acquisition was performed in non-overlapping ~500-turn enameled wire (30 AWG) coils supported in closed plastic containers (inner dimensions, 11 cm diameter × 14 cm tall) with both terminals of each coil connected to a phono preamplifier (Pyle PP444). The amplified signal output was recorded at a 1000 Hz sampling rate using a myDAQ (National Instruments) data acquisition system controlled through MATLAB (Mathworks, R2018a version, along with the NI myDAQ support package). The analog input range for each channel was ±10 V with an ADC (analog-digital conversion) of 16 bits. The amplified signal amplitude rarely exceeded a ±2.5 V range which eliminated the risk of signal clipping. In total, we were able to perform the simultaneous recording of 4 coils per session in two data acquisition systems (2 coils per myDAQ unit), but these number can be scaled up by using additional data acquisition systems, or systems with more analog input channels.

Visual identification of HTR was performed as previously described^5^ on clips recorded at 60 FPS and 1080p resolution. For visual identification of HTR as signals on the magnetometer recorded data, the raw data was processed on the MATLAB suite. The recording was band-passed through a digital Butterworth filter between 40–200 Hz as previously described^17^, and the filtered data plotted in parallel with a spectrogram heat-map. HTR were identified when: i) amplitude of the voltage signal exceeded the background noise level, and ii) corresponded in time with a peak maximum in the region of 70–110 Hz in the spectrogram heat-map often accompanied by a harmonic between 40–60 Hz of comparable or greater magnitude.

Automated detection of HTR events relied on a script based on detection of individual events between 70–110 Hz. Recorded data was band-passed through a digital Butterworth filter between 70–110 Hz and transformed to absolute values. After double local maxima processing each original wavelet is transformed into a unipolar peak that is identified as HTR event when (i) peak prominence exceeds 0.075 V, (ii) is separated from any other event by at least 200 ms, and (iii) has a width inferior to 90 ms at half the maximum value of prominence. The full script can be found in the Supplementary Material section.

Experiments that included the recording of the piezo sensor output were carried out with a paper tube (46 cm height) surrounding the coil and open top so that the animal could jump vertically without escaping the container. Simultaneous recording of the coil and piezo sensor signal was done by connecting the amplified output of the coil to one analog input channel of the data acquisition system (as described above), and the output of the piezoelectric sensor (ceramic diaphragm, 27 mm brass disc) secured under the plastic container with tape directly into the other available analog input channel. The recording data from the piezo sensor baseline was corrected and the signal transformed to absolute values before performing a maxima analysis in the MATLAB environment. Employing the peak-picking function timestamps for maxima exceeding an absolute voltage of 0.3 V were annotated. The value for the 0.3 V threshold was determined experimentally as described above, and corresponded to ~8 standard deviations of the piezo sensor signal from mice injected with 1 mg/kg of DOI (Fig. 3C). The matching analysis of HTR detection and annotated piezo sensor maxima timestamps was automated. Two events were matched if the HTR detection system timestamp fitted within the range defined by the piezo sensor maxima timestamp ±0.1 s.

Analytical reference (±)-2,5-dimethoxy-4-iodoamphetamine, 2,5-dimethoxy-4-bromophenethylamine were purchased from Cerilliant Corporation (Round Rock, TX). Acetonitrile, ammonium acetate, ethyl acetate, formic acid, hexane, methanol, sodium hydroxide and water were purchased from Fisher Scientific (Hanover Park, IL, USA). All reagents were ACS grade or higher.

### Ultra high-performance liquid chromatograph tandem mass spectrometer (UHPLC-MS/MS) analysis of DOI in mouse whole blood and forebrain

After the administration of DOI (1 mg/kg, i.p.), mice were sacrificed by cervical dislocation at different time-points. The whole blood was collected immediately after decapitation in a vial containing 1 mg EDTA (aq.). After extraction of the brain, the forebrain was dissected by mechanical separation of the midbrain. The whole blood, brain tissue samples and quality control specimens prepared in mouse blood and brain homogenate (1:3 tissue: water) at low (3 ng/mL or g), mid (30 ng/mL or g) and high (75 ng/mL or g) concentration of DOI were kept at –20 °C until analyzed. On the day of analysis, seven-point calibration curves at concentrations of 1, 5, 10, 20, 100, 200 and 500 ng/mL blood or ng/g brain tissue of DOI along with a drug free control and a negative control without internal standard (10 ng/mL or g 2,5-dimethoxy-4-bromophenethylamine (2-CB)) in drug-free serum were prepared. The quality controls were aliquoted in triplicate with each analysis. DOI was extracted from the blood and tissue using a modified previously published liquid/liquid extraction for the analysis of 4-bromo-2, 5-dimethoxy-*N-*[(2-methoxyphenyl) methyl]-benzeneethanamine^53^. In brief, 1 ng of 2-CB, the internal standard (ISTD), was added to 100 μL aliquots of blood or 400 μL of brain homogenate of each calibrator, control or specimen except the negative control. Then 100 μL of 0.2 M sodium hydroxide solution was added followed by 0.5 mL of hexane/ethyl acetate (9:1). The samples were mixed for 5 min and then centrifuged for 10 min at 3000 rpm. The upper organic layer was transferred to a clean test tube and evaporated to dryness under a gentle stream of nitrogen in a 40 °C dry bath. The samples were reconstituted with 100 μL of mobile phase and placed in auto-sampler vials for analysis.

The ultra high-performance liquid chromatograph tandem mass spectrometer (UHPLC-MS/MS) analysis of was performed on a Sciex 6500 QTRAP system with an IonDrive Turbo V source for TurbolonSpray® (Sciex, Ontario, Canada) attached to a Shimadzu UPLC system (Kyoto, Japan) controlled by Analyst software (Sciex, Ontario, Canada). Chromatographic separation was performed on a Hypersil Gold C8 100 × 2.1 mm, 3 *μ* column (Thermo Scientific, Waltham, MA, USA). The mobile phase consisted of A: Water with 10 mM ammonium acetate and 0.1% formic acid and B: Acetonitrile. The following gradient was applied: 0.00–0.10 min, 20% B, a linear gradient to 70% B at 3.00 min, hold for 0.90 min, then return to 20% B at 4.0 min. The source temperature was set at 650 °C and had a curtain gas flow rate of 30 mL/min. The ionspray voltage was 5000 V, with the ion source gases 1 and 2 at flow rates of 25 mL/min. The acquisition mode used was multiple reaction monitoring (MRM). The retention times were: DOI, 2.85 min; and 2-CB, 2.64 min. The declustering potential was set at 80 eV. The following transition ions (m/z) were monitored in MRM mode with their corresponding collection energies (eV) in parentheses: DOI: 322 > 305 (15) and 322 > 271 (28); and 2-CB: 260 > 243 (15) and 260 > 228 (28). The total run time for the analytical method was 5 min. A linear regression of the peak area of ratios of the quantification and the ISTDs transition ion were used to construct the calibration curves.

The method was evaluated for linearity, lower limit of quantification (LOQ), lower limit of detection (LOD), bias, precision, carryover, ion suppression and selectivity. Linearity was verified from seven-point calibration curves at concentrations of 1, 5, 10, 20, 100, 200 and 500 ng/mL blood or ng/g brain tissue of DOI. The linear regression correlation coefficients (R square) for the all the DOI calibration curves were 0.9960 or better. The LOD and LOQ were administratively set at 1 ng/g and were determined to be ±20% of the target concentration and had responses at least ten times greater than the signal to noise ratio of either the DOI free blood or brain tissue homogenate. Bias and precision were determined using the prepared quality control specimens. The determined bias with the precision expressed as % coefficient of variation (%CV) in parentheses for blood were 12 (7), −4 (6) and 11 (8) for the LQC, MQC and HQC, respectively and for brain were 15 (2), −12 (7) and 4 (1) for the LQC, MQC and HQC, respectively. Sample carryover was evaluated by analyzing a negative control immediately after the analysis of the high calibrator. Lack of carryover was confirmed as DOI was not detected in the negative control. The post-extraction addition approach was used to assess the matrix effects (n = 3) for blood and brain homogenate at 10 ng/mL or g for DOI and 2-CB, the ISTD. The matrix effect was determined to be 6 ± 13 for DOI and 5 ± 13 for 2-CB in blood and 9 ± 36 for DOI and 24 ± 37 for 2-CB in brain homogenate. The selectivity of the assay was determined analyzing six different lots of DOI free blood and brain homogenate. Each individual lot was analyzed with and without ISTD. No peaks were detected that co-eluted with the DOI or with the ISTD. This ensured that endogenous brain homogenate components did not interfere with the assay.

### Statistical analysis

Statistical analyses were performed with GraphPad Prism software version 8. Animals were randomly allocated into the different experimental groups. Experiments combining DOI and mGluR2 ligands were performed crossing groups with 1 week between tests. Statistical significance of experiments involving different treatment groups were assessed by one-way followed by Bonferroni’s *post hoc* test, and in experiments involving two or more variables and treatment groups by two-way ANOVA followed by Bonferroni’s *post hoc* test. Statistical significance of experiments involving two treatment groups was assessed by Student’s *t*-test. The level of significance was chosen at *P* < 0.05. All data are presented as mean ± standard error of the mean (S.E.M). Correlation between visual identification vs. automated detection of HTR, and visual analysis of signal vs. automated detection of HTR were assessed by linear regression. Correlation between levels of DOI in whole blood, forebrain and HTR was assessed by Pearson’s correlation coefficient.

## Supplementary information

Matlab script

Supplementary Figures

## Data Availability

The data from this study are available from the corresponding author upon reasonable request.
