# Supplementary material for: Fully automated head-twitch detection system for the study of 5-HT2A receptor pharmacology in vivo
Source: Sci Rep. 2019 Oct 3;9:14247. doi: 10.1038/s41598-019-49913-4 (PMC6776537; doi:10.1038/s41598-019-49913-4)
Supplement: Supplementary file 2 — Supplementary Figures [file 41598_2019_49913_MOESM2_ESM.pdf]

# Fully-automated head-twitch detection system for the study of 5-$HT_{2A}$ receptor pharmacology in vivo

Mario de la Fuente Revenga, Jong M. Shin, Hiba Z. Vohra, Kelsey S. Hideshima, Matthew Schneck, Justin L. Poklis and Javier González-Maeso

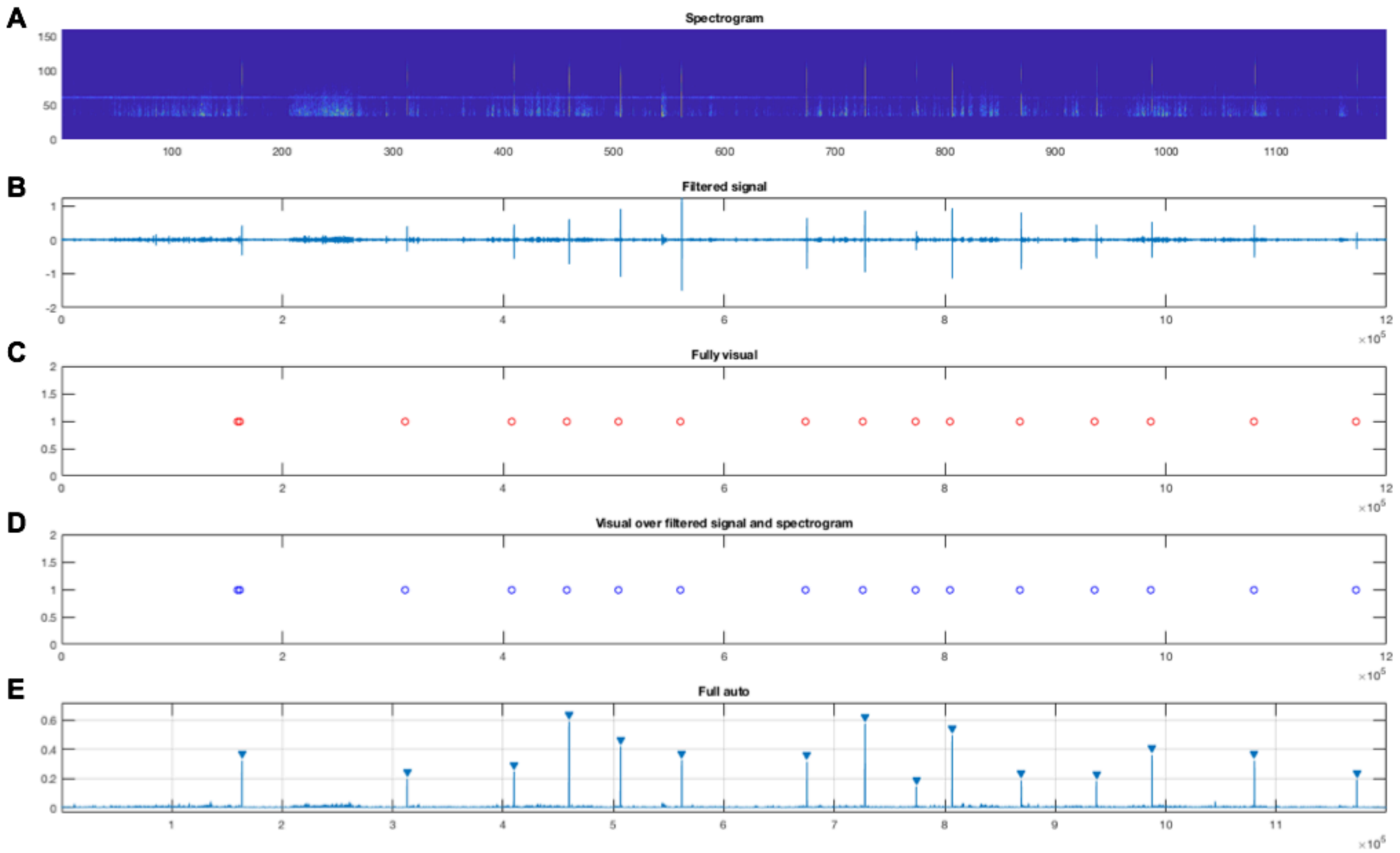


**Figure S1.** (**A**) Spectrogram heat-map from 0 to 150 Hz of (**B**) 40-200 Hz band-pass filtered data of a control mouse injected with DOI 1 mg/kg (i.p.). (**C**) HTR detected by direct observation of the animal (red circles). (**D**) HTR identified by analyzing the voltage amplitude signal (**B**) and its corresponding spectrogram (**A**) (blue circles). (**E**) Signal processed by the automated HTR detection system and identified HTR events (blue triangles). **A**, frequency in the *y*-axis (Hz), time in the *x*-axis (s). **B-E**, voltage in the *y*-axis (V), time in the *x*-axis (ms). The faint blue band around 60 Hz corresponds to noise from the AC power supply.

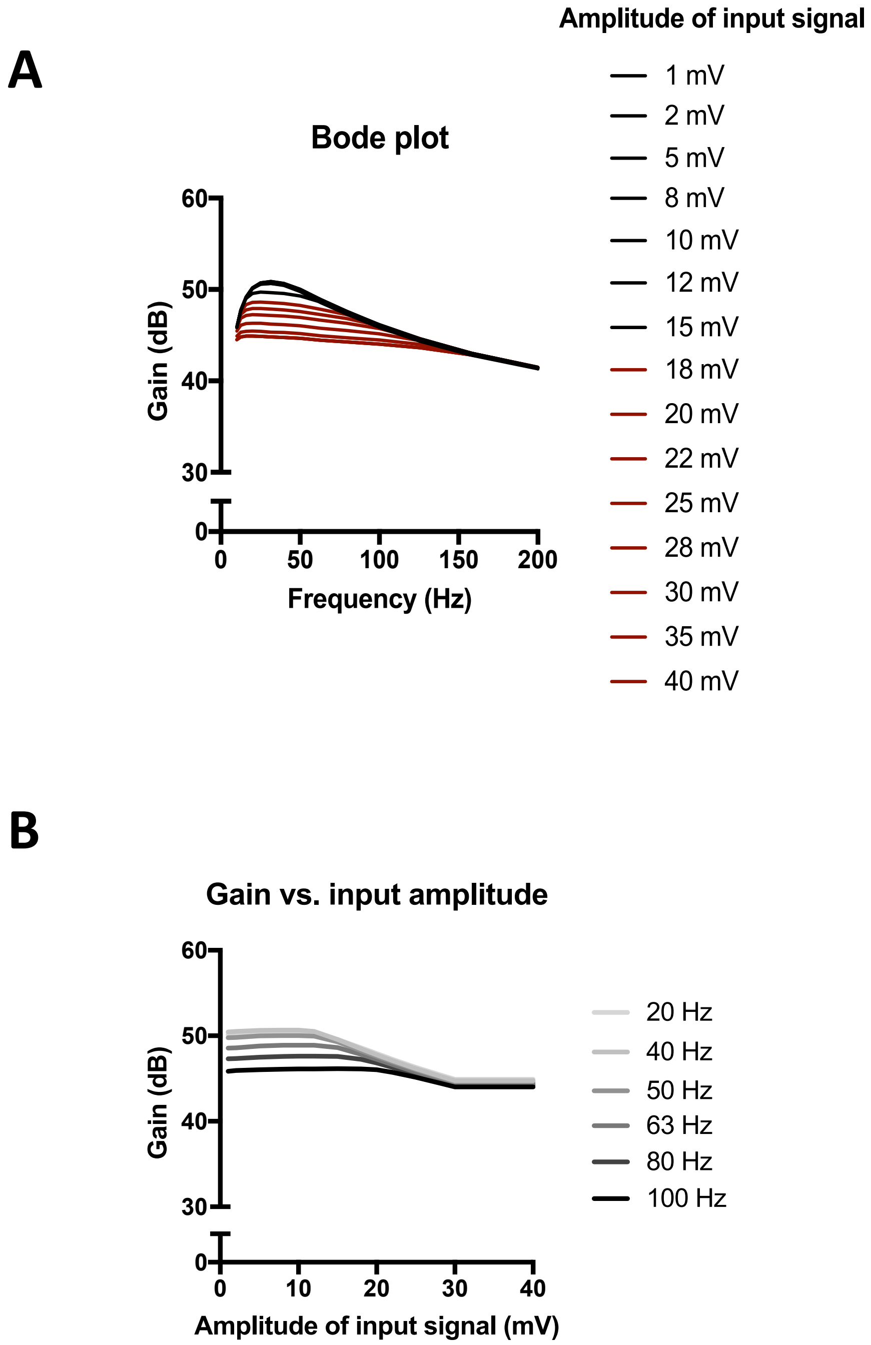


**Figure S2.** (**A**) Gain as a function of frequency (1—200Hz) for input signals of varying amplitudes. For clarity, the traces corresponding to amplitudes of 15 mV or below are colored in black and the traces above 15 mV in red. (**B**) Gain as a function of amplitude in the input signal for a selected frequencies from panel A.

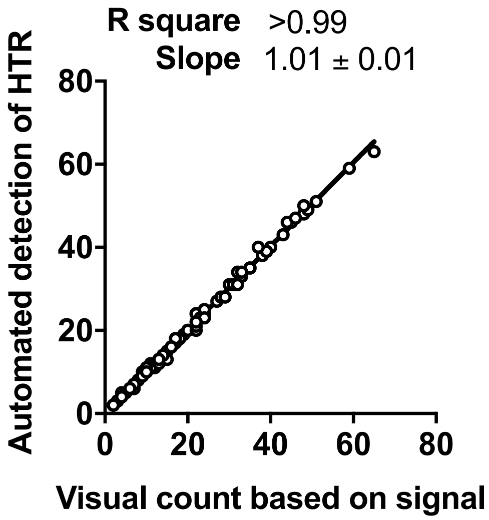


**Figure S3.** Correlation between automated detection and visual count of HTR based on signal analysis of 100 data sets of 10-week-old control mice treated with different doses of DOI.

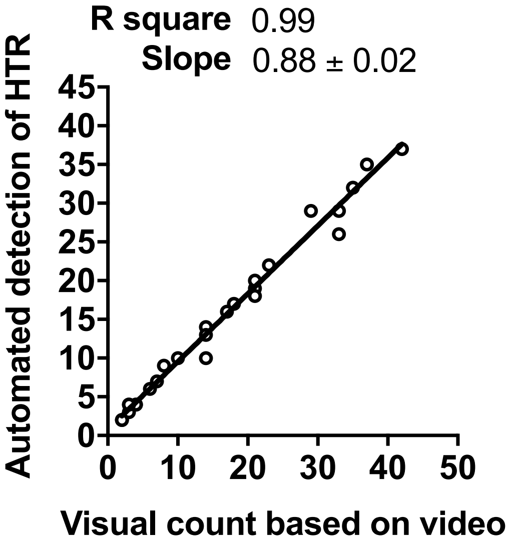


**Figure S4.** Correlation between automated detection and visual count of HTR on ~12-month-old control mice treated with different doses of DOI.

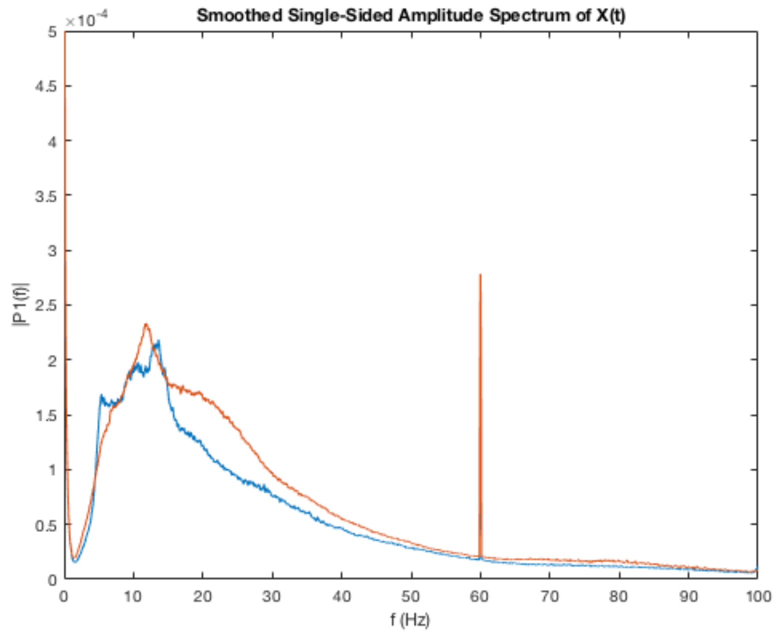


**Figure S5.** Averaged periodograms of raw 90 min recording from control mice administered with MK801 (0.5 mg/kg, orange trace, n = 6) or vehicle (blue trace, n = 6). Power in the *y*-axis, and frequency in the *x*-axis (Hz). The peak observed at 60 Hz corresponds to noise from the AC power supply.

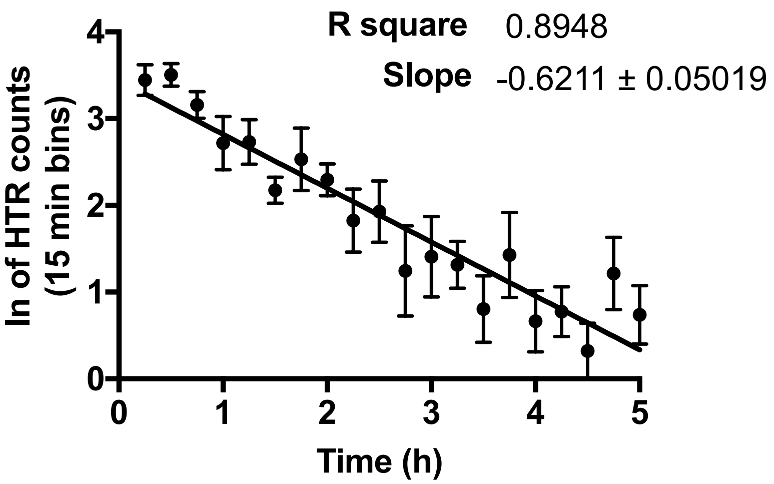


**Figure S6.** Natural logarithm transformation of HTR-induced by DOI (1 mg/kg) in 15 min bins from Figure 4A. Each time point represents the mean of the natural logarithm ± S.E.M. Linear regression of the natural logarithm (*y*-axis) relative to time (*x*-axis). Slope value ± S.E.M.

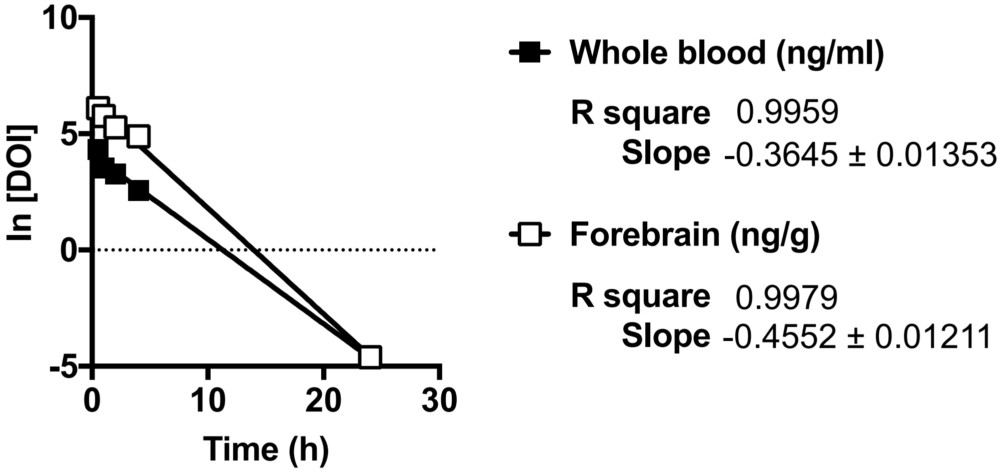


**Figure S7.** Natural logarithm transformation of the concentration of DOI in whole blood and forebrain from Figure 4B. Each time point represents the mean of the natural logarithm. The S.E.M. are smaller than the symbols and have been omitted. Linear regression of the natural logarithm (*y*-axis) relative to time (*x*-axis). Slope value ± S.E.M.

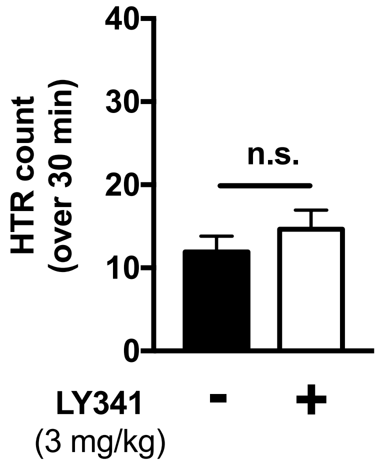


**Figure S8.** Total HTR count of control mice treated with LY341495 (3 mg/kg, i.p.) or vehicle over a period of 30 min after the administration of the drug. Bars represent mean ± S.E.M. Two-tailed Student t-test (n.s., not significant)
